# Optimization of MAE for the Separation of Nicotine and Phenolics from Tobacco Waste by Using the Response Surface Methodology Approach

**DOI:** 10.3390/molecules26144363

**Published:** 2021-07-19

**Authors:** Marija Banožić, Ines Banjari, Ivana Flanjak, Mate Paštar, Jelena Vladić, Stela Jokić

**Affiliations:** 1 Faculty of Food Technology Osijek, Josip Juraj Strossmayer University of Osijek, Franje Kuhača 18, 31000 Osijek, Croatia; marija.banozic@ptfos.hr (M.B.); ines.banjari@ptfos.hr (I.B.); ivana.flanjak@ptfos.hr (I.F.); 2 Public Institution RERA S.D. for Coordination and Development of Split-Dalmatia County, Domovinskog rata 2, 21000 Split, Croatia; mate.pastar@rera.hr; 3 Faculty of Technology, University of Novi Sad, Bulevar cara Lazara 1, 21000 Novi Sad, Serbia

**Keywords:** tobacco waste, microwave-assisted extraction, optimization, bioactive compounds

## Abstract

This study intends to valorize by-products of the industrial processing of tobacco to obtain nicotine and phenolics as value-added compounds. Three influential parameters of the microwave-assisted extraction-MAE (temperature, treatment time, and solvent/solid ratio) were studied for the optimization of the extraction protocol for tobacco leaves and three types of waste—scrap, dust, and midrib, respectively. Nicotine was the dominant bioactive compound in all extracts, ranging from 1.512 to 5.480% in leaves, 1.886 to 3.709% in scrap, 2.628 to 4.840% dust, and 0.867 to 1.783% in midrib extracts. Five phenolic compounds were identified and quantified, predominated by chlorogenic acid and rutin. Additionally, total phenol content and antioxidant activity were determined using spectrophotometric assays. Optimization was performed in two aspects: to obtain a maximum extraction yield with minimum nicotine content and to obtain a maximum extraction yield with maximum nicotine content. These findings demonstrate that tobacco waste is a valuable source of bioactive compounds and MAE can be a promising alternative technique to obtain extracts rich in targeted bioactive compounds, especially nicotine.

## 1. Introduction

Tobacco is an industrial non-food crop, containing large amounts of high added-value compounds, such as alkaloids (mainly nicotine), phenolic compounds (chlorogenic acids and rutin), carbohydrates, and aroma compounds [1]. At present, large quantities of tobacco parts are discarded as waste products from the cropping, handling, processing and consuming of tobacco and tobacco products. Tobacco industry waste is a processing by-product, available in large amounts across the world, and impropriate for disposing due to the high content of nicotine [2,3]. Separated and purified nicotine could be used in nicotine replacement therapies such as gums and patches. Moreover, after extraction of nicotine, tobacco waste becomes more appropriate for using as fertilizer, fiberboards, and pulps [4]. Moreover, reducing of nicotine content is important for producing new tobacco non-addictive products. Current industrial strategies for tobacco waste are based on low-economic income solutions, such as production of the reconstituted tobacco [5]. The quantity of waste generated during tobacco production and processing, combined with the beneficial characteristics of bioactive compounds present in these wastes, justifies the great interest of researchers in the extracting of valuable compounds from tobacco waste. Therefore, different approaches and techniques were suggested, including supercritical fluid extraction [3,6], subcritical water extraction [7], and high-voltage electric discharge assisted extraction [8]. Moreover, conventional extraction methods, using common organic solvents, were proven to be efficient in the separation of bioactive compounds from tobacco waste while several studies used microorganisms to reduce nicotine content in tobacco waste [9,10]. However, safety issues of the final products favor green extraction techniques over conventional [11].

Recently, microwave energy has been used in analytical chemistry for sample preparation, digestion, synthesis, and for extraction processes [12,13]. Microwave-assisted extraction (MAE) is a process of using microwave energy to heat solvents and enhance the penetration of solvents into the plant material [14]. There are several mechanisms that can influence the mass transfer during MAE and they are based on ionic conduction and dipole rotation of molecules [15]. The main advantages of MAE over conventional extraction techniques are the reduced treatment time, the reduced solvent consumption, the higher extraction yield, and enhanced quality of the obtained extracts [16]. Application of the MAE has been rising rapidly in the last two decades, especially in the separation of bioactive compounds [17,18]. There are several papers dealing with MAE of bioactive compounds from tobacco leaves and waste. Those studies mostly used an open vessel system and obtained non-polar fractions where different volatile compounds were determined. Zhu et al. [19] used MAE for the separation of volatile organic acid from tobacco leaves. Joners et al. [20] extracted tobacco leaves alkaloids using microwave energy while Nie et al. [21] used MAE with deep eutectic solvent combined with solid-phase microextraction for volatile compounds separation. Moreover, two studies dealt with MAE of the solanesol from tobacco-related materials [22,23]. Only one study was conducted for the separation of polar fractions where Li et al. [24] used MAE coupled with capillary zone electrophoresis as a pretreatment for the fast determination of the chlorogenic acid in tobacco waste samples. To the best of our knowledge, the systematic research on MAE of polar bioactive compounds from different types of tobacco waste was not performed.

The present work describes the efficiency of MAE for recovery of targeted bioactive compounds from tobacco industry waste. The effects of various extraction parameters including temperature, extraction time and solvent/solid ratio were optimized. Obtained results were compared with tobacco leaves as material origin. That comparison provided detail evaluation of MAE on tobacco waste as base for the most rational valorization of tobacco waste. On the basis of experimental results, this study proposed quadratic and cubic mathematical models to predict extraction efficiency.

MAE of nicotine may be observed from two aspects: as the method for obtaining low-nicotine extracts, rich in phenolic compounds or as a method for obtaining extracts rich in nicotine where after extraction nicotine-exhausted waste material is obtained, which can later be used for other purposes or safer disposing.

For this purpose, the provided research aims to be a useful engineering tool for development of green extraction process of bioactive compounds from tobacco waste, considering the diversity of each type of tobacco waste.

## 2. Results and Discussion

MAE was applied at different temperatures (80–120 °C), times (5–25 min), and solvent/solid ratios (300–700 mL/g), and resulting in 17 combinations of these variables for leaves and each tobacco waste type (scrap, dust, and midrib). The objective of the present study was to determine the potential of MAE from tobacco waste compared to MAE from leaves, and also to determine the influence of process parameters, and optimize the nicotine extraction process.

### 2.1. Bioactive Compounds Composition of Tobacco Leaves and Waste Extracts Obtained with MAE

MAE is well known for accelerating the extraction process and improving the extraction yield [12,14]. In this study, extraction yield was in the range from 19.40 to 69.78% for tobacco leaves, from 35.98 to 69.08% for scrap, from 29.42 to 58.23% for dust, and from 37.84 to 57.96% for midrib (Table 1, Table 2, Table 3 and Table 4). For all waste samples, the highest extraction yield was obtained at temperature of 120 °C. During MAE microwave power was dependent on temperature. From Supplementary Materials Figure S1, it can be seen that when the temperature was set at 80 °C, the microwave power reach its maximum (700 W) first 2 min, then decreased on the value of 100 W during the extraction process while on 120 °C microwave power reach maximum (700 W) at first 4 min, then decreased on the value of 200 W during the extraction process. Thus, the higher extraction yield showed to be directly related to the higher microwave power applied.

In comparison with our previously published results, for the subcritical water extraction [7] and two-stage extraction process (supercritical and subcritical extraction) [25] the obtained extraction yields were slightly lower. Furthermore, in comparison with results for ultrasound-assisted extraction obtained results were slightly higher [26]. Since, ultrasound-assisted extraction was performed on lower temperatures (up to 70 °C), higher applied temperatures could be responsible for increase of extraction yield. On the other hand, obtained results were significantly higher than those obtained with supercritical extraction [25]. However, subcritical water extraction was conducted at significantly higher temperatures (150–250 °C) while supercritical CO_2_ extraction was highly selective to non-polar compounds which are naturally present in very low concentrations in tobacco materials. Moreover, obtained yields were significantly higher in comparison with the conventional extraction process performed by Docheva et al. [27] where for Virginia blend type lower extraction yield (34%) was reported.

The obtained results for the extracted amount of bioactive compounds from tobacco leaves and waste are summarized in Table 1, Table 2, Table 3 and Table 4. In this study nicotine was dominant compound with concentrations in range 1.512–5.480, 1.886–3.709, 2.628–4.840, and 0.867–1.783% for leaves, scrap, dust, and midrib extracts, respectively. Nicotine well known as dominant bioactive compound of tobacco, naturally present in a concentration from 0.3 to 3% referred to dry plant material [28].

Moreover, the concentration of nicotine is highly dependent on tobacco type, variety, growing and environmental conditions [6,29]. In comparison to other extraction techniques, nicotine concentrations were similar to those obtained with subcritical water extraction. However, concentrations for leaves and dust were slightly higher than those obtained with subcritical water extraction. Yet, those differences should be carefully interpreted because nicotine tends to degrade under the conditions of subcritical water extraction [7].

A recent study by Popova et al. [30] also found that nicotine was a major compound in tobacco resinoid (extract obtained with polar solvent), where nicotine concentrations were over 3%. Additionally, a spectrophotometric assay for determination of antiradical activity was performed. Applied process parameters did not significantly influenced the antiradical activity of MAE extracts, while obtained values were similar to those obtained for tobacco waste with other extraction techniques [7,8,26].

Besides nicotine (alkaloid), five phenolic compounds were detected in leaf extracts, namely, chlorogenic acid (CA), neochlorogenic acid (NCA), cryptochlorogenic acid (CCA), rutin, and nicotiflorin (Table 1, Table 2, Table 3 and Table 4). Polyphenols profile of leaves extract were in conformity with the findings from other studies [2,29] where other extraction techniques were applied. In comparison with leaf extracts, waste extracts were less rich in bioactive compound content implying possible losing of the bioactive compounds during tobacco processing which was also confirmed in our previous studies [7,26]. CA (5-*O*-caffeoylquinic acid), NCA (3-*O*-caffeoylquinic acid), and CCA (4-*O*-caffeoylquinic acid) are members of the phenolic acid group named chlorogenic acids and represent the main phenolic acids in tobacco [31]. CA was present in concentrations in range 0.145–1.501, 0.180–0.525, 0.237–1.135, 0.014–0.265% in leaves, scrap, dust and midrib extracts, respectively. NCA concentrations varied in range 0.054–0.409, 0.048–0.256, 0.119–0.344, 0.055–0.323% in leaves, scrap, dust and midrib extracts, respectively. CCA acid concentration were in range 0.017–0.432, 0.030–0.136, 0.012–0.249, and up to 0.444% in leaves, scrap, dust and midrib extracts, respectively. Chlorogenic acid contents were in good agreement with a study from Wang et al. [2] where sonification was used as an extraction technique. Moreover, two dominant flavonoids were identified, rutin (quercetin 3-rutinoside) and nicotiflorin (kaempferol-3-*O*-rutinoside). Both are rutinosides group members. In this study, rutin concentrations were in range 0.111–0.635, 0.130–0.631, 0.126–0.602, 0.079–0.166% in leaves, scrap, dust and midrib extracts, respectively. Nicotiflorin concentrations were up to 0.089% for leaves, and from 0.040 to 0.083% in scrap, from 0.039 to 0.099% in dust and up to 0.075% in midrib. The content of individual phenolic compounds was similar to those obtained in the study [29] for Plovdiv variety.

### 2.2. Analysis of Response Surface Plots

**Figure 1:**
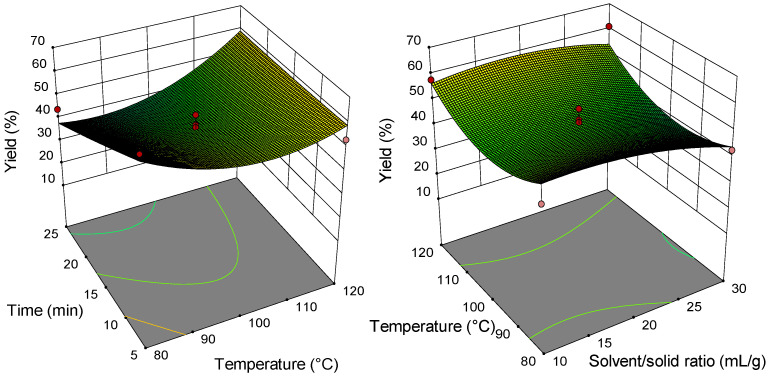
Three-dimensional (3D) plot showing the combined effects of MAE process condition on extraction yield of tobacco leaves.

**Figure 2:**
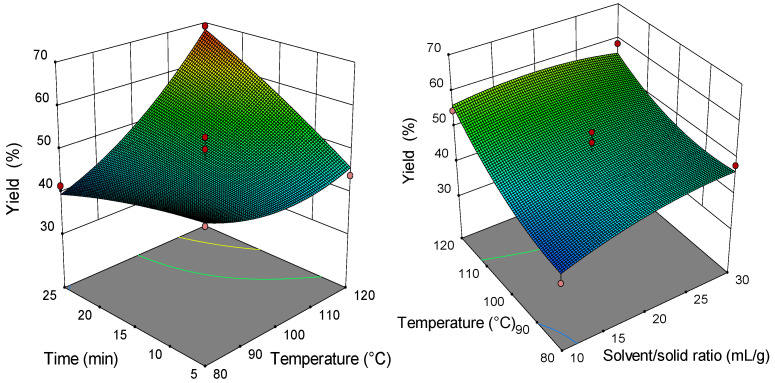
The 3D plot showing the combined effects of MAE process condition on extraction yield of tobacco waste (type: scrap).

**Figure 3:**
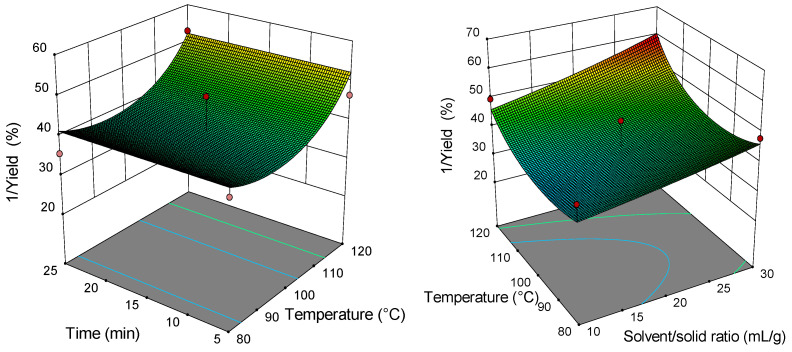
The 3D plot showing the combined effects of MAE process condition on extraction yield of tobacco waste (type: dust).

**Figure 4:**
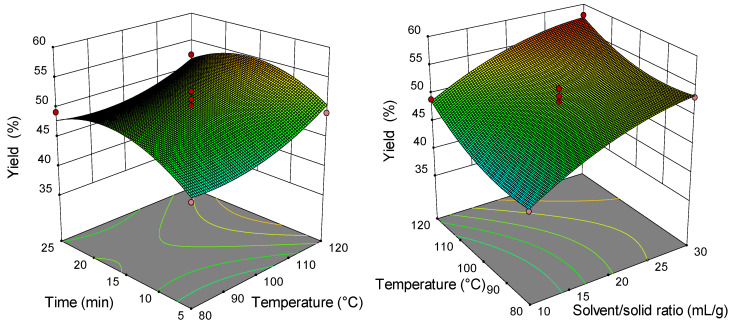
The 3D plot showing the combined effects of MAE process condition on extraction yield of tobacco waste (type: midrib).

**Figure 5:**
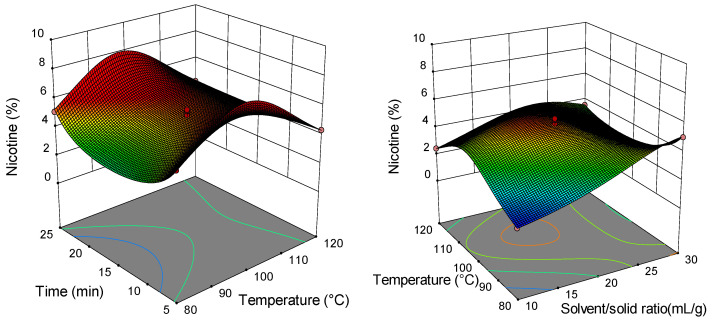
The 3D plot showing the combined effects of MAE process condition on nicotine content in tobacco leaves.

**Figure 6:**
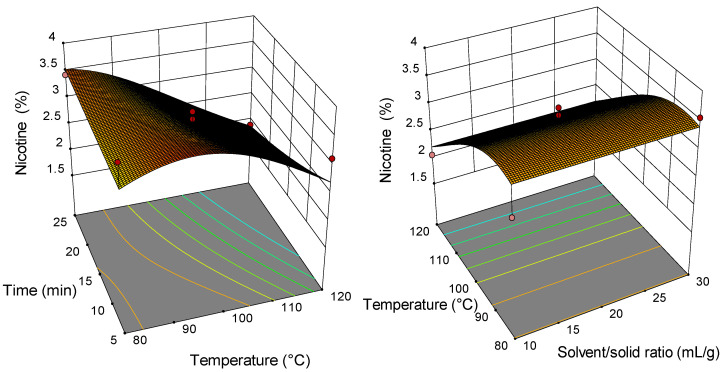
The 3D plot showing the combined effects of MAE process condition on nicotine content in tobacco waste (type: scrap).

**Figure 7:**
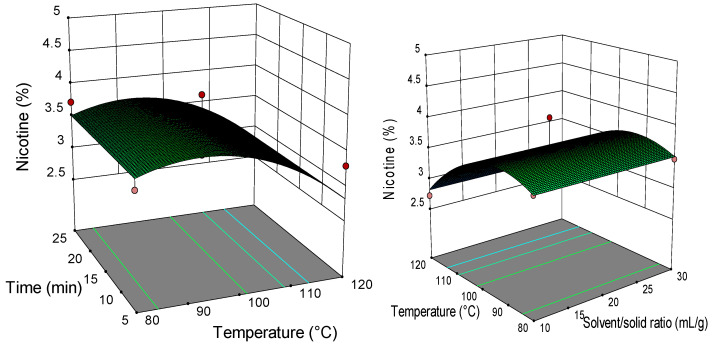
The 3D plot showing the combined effects of MAE process condition on nicotine content in tobacco waste (type: dust).

**Figure 8:**
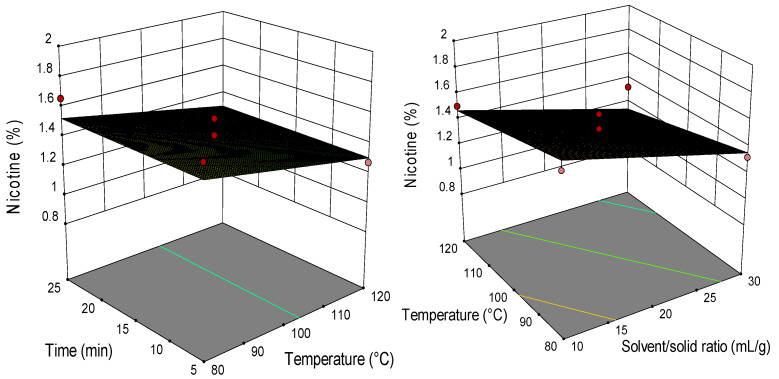
The 3D plot showing the combined effects of MAE process condition on nicotine content in tobacco waste (type: midrib).

Three-dimensional (3D) response surfaces are constructed to give a visual interaction between two parameters and facilitate the optimal conditions for maximal response data (Figure 1, Figure 2, Figure 3, Figure 4, Figure 5, Figure 6, Figure 7 and Figure 8). From all 3D plots, it can be seen that extraction efficiency (extraction yield) increases with increasing of temperature. Since microwave power was dependent on temperature (temperature is set as a maximum value) on higher temperatures, higher energy was supplied and diffusion process between solid and solvent become faster. Similar was reported in the study by Kamaruddin et al. [32] where prolonged extraction time did not significantly influence the extraction yield. Hu et al. [33] even reported that extraction efficiency can decrease when the irradiation time is longer than 4 min. Those phenomena is usually related to the decomposition of sensible compounds during prolonged irradiation. Solvent/solid ratio showed a different effect on different types of tobacco waste. In the case of leaves and dust, extraction yield slightly decreases with increase of solvent/solid ratio while for scrap, and midrib extraction yield slightly increased with a higher solvent/solid ratio. The positive influence of solvent/solid ratio can be explained with the relation between moisture content and microwave irradiation. Namely, when the moisture level in material increases, the number of water-containing plant cells increases as well. Therefore, cells are ruptured and a higher extraction yield is obtained. The negative influence of solvent/solid ratio can be related to the inappropriate stirring of the larger volume of solvent. Alara et al. [34] reported that a larger volume of solvent can require more absorption of microwave energy and result in insufficient energy for the cell wall breakage. Therefore, the influence of solvent/solid ratio is dependent on the plant material and which phenomena will be dominant.

### 2.3. Statistical Analysis

For better understanding of differences between waste and leaves, as well as influence of process condition on MAE, statistical analysis was provided (Supplementary Materials). As Table S1 shows, for leaves, higher yield correlates significantly with nicotiflorin and total phenols. For scrap, higher yield means less rutin, and the same negative correlations can be seen for dust. The impact of extraction yield is more evident on dust samples, where higher yield provides significantly lower amounts of nicotiflorin, nicotine, rutin and lower radical scavenging activity (DPPH). This can be again explained through correlation between temperature and microwave power, explained previously. With the increase of temperature above 100 °C, higher temperatures caused an extraction yield enhancement for all samples. On the other hand, increase of temperature means waste of energy and lower content of bioactive compounds. 

Sample comparison was done between tobacco leaves (chosen as a model sample), as starting material with all other samples (tobacco waste materials). Differences in yield selected components concentrations and antiradical activity (DPPH) are shown in Table S2. As expected, the biggest difference was found between leaves and midrib. This is probably due to physiological and chemical differences between leaf lamina and midrib previously explained by Banožić et al. [1]. The midrib is the central vein of a leaf and has more malonic and oxalic acids, total ash, calcium, potassium and chlorine while leaf lamina has more reducing sugars, total alkaloids, nitric and citric acid, and phenols. Interestingly, yield does not significantly differ between leaves and scrap which can again be explained with materials similarity since scrap is more similar to leaf than other types of waste.

Finally, the comparison between applied process parameters was done based on the extraction method. The comparator method used to compare all others was 100 °C, 15 min and 20 mL/g of solvent/solid ratio, and central point of Box–Behnken design (BBD). Method #1 was 80 °C, 5 min and 20 mL/g of solvent/solid ratio, method #2 80 °C, 25 min and 20 mL/g of solvent/solid ratio, method #3 120 °C, 5 min and 20 mL/g of solvent/solid ratio, and method #4 120 °C, 25 min, 20 mL/g of solvent/solid ratio. The only difference was found between Comparator and Method #4 where Comparator gave higher yield (*p* = 0.004).

### 2.4. Predictive Modeling and Optimization of MAE of Bioactive Compounds from Tobacco Waste

In order to obtain the regression models, the experimental data (responses) were analyzed and fitted to various models (quadratic and cubic). Obtained models represent an empirical relationship between dependent variables (responses) and independent variables (process condition). Models are expressed by a second-order polynomial equation with interaction terms, which serves for the extraction efficiency prediction:

Y_L(Yield)_ = 45.28 − 7.21X_2_ + 8.58X_1_^2^ + 7.29X_1_X_2_ − 9.27X_2_X_3_ (1)

1/Y_L(Nicotine)_ = 0.20 − 0.043X_1_ + 0.033X_3_ + 0.082X_1_^2^ − 0.07X_2_^2^ + 0.10X_3_^2^ + 0.083X_1_X_3_ − 0.032X_2_X_3_ − 0.17X_1_^2^X_3_ + 0.063X_1_X_2_^2^ (2)

Y_S(Yield)_ = 48 + 6.69X_1_ + 3.51X_2_ + 7.69X_1_X_2_ (3)

1/Y_S(Nicotine)_ = 0.32 − 0.078X_1_ + 0.035X_2_ + 0.054X_1_^2^ + 0.047X_1_X_2_ (4)

1/Y_D(Yield)_ = 0.026 − 2.498 × 10^−3^X_1_ − 2.534 × 10^−3^X_3_ − 3.735X_1_^2^ + 2.772 × 10^−3^ X_2_X_3_ (5)

Y_D(Nicotine)_ = 3.61 − 0.34X_1_ − 0.45X_1_^2^ − 0.54X_2_X_3_ (6)

Y_M(Yield)_ = 50.49 + 2.50X_1_ + 4.84X_3_ − 3.85X_2_^2^ (7)

Y_M(Nicotine)_ = 1.41 − 0.11X_1_ − 0.17X_3_ + 0.24X_1_X_3_ (8)

where X_1_, X_2_, and X_3_ are the coded variables for temperature, time, and solvent/solid ratio, respectively.

The fitting Equations (1)–(8), which describe the dependence of extraction yield and nicotine content on the independent variables (temperature, time and solvent/solid ratio) were used for the individuation of the optimal extraction operating conditions. The optimization was carried out in two aspects; to obtain a maximum extraction yield with minimum nicotine content and secondly, to obtain a maximum extraction yield with maximum nicotine content. Optimization results are showed in Table 5. Theoretical results were experimentally confirmed with an average relative deviation of up to 5%. This reveals that proposed models have high predictive ability for the optimization of the MAE process. In comparison with leaves, it can be seen that the obtained nicotine values in waste are significantly lower. Even the waste is directly delivered from leaves, it is subjected to various other processes after separation of leaf lamina [1,35]. Moreover, from Table 5, it can be seen that the higher difference in obtained nicotine values are in midrib extracts, where, also, a higher treatment time as optimum was provided. Those results clearly demonstrate that every type of tobacco waste should be consider separately, according to its unique properties and chemical composition of each type of tobacco waste.

## 3. Materials and Methods

### 3.1. Plant Material

Tobacco leaves-commercial tobacco blend Virginia (consisted of 26.7% Oriental tobacco and 73.3% Virginia tobacco) and waste samples (scrap, dust, and midrib) derived from the same blend were collected in August 2020 from tobacco processing factory “Fabrika duhana Sarajevo”, Sarajevo, Bosnia and Herzegovina. The samples were ground with an MRC Sample mill (C-SM/450-C, Holon, Israel) to obtain a homogenous drug powder. The drug material was sieved and fraction with particle size less than 2 mm was stored in dark conditions at +4 °C until extraction. The humidity value of the sample did not change during the storage.

### 3.2. Chemicals and Standards

All HPLC solvents were from J.T. Baker (Phillipsburg, NY, USA) and were HPLC grade. The ethanol (99.9%) used for extraction procedure and other used chemicals were an analytical grade. Standard of nicotine (purity: 98.40%), and chlorogenic acid (purity: 97.2%) were obtained from Dr. Ehrenstorfer GmbH (Augsburg, Germany) while standards of cryptochlorogenic (purity: ≥98%) and neochlorogenic (purity: 98%) were obtained from Sigma-Aldrich (Steinheim, Germany). The standard of rutin (purity: 97%) was purchased from Acros Organics (Geel, Belgium) and nicotiflorin (purity: ≥95.0%) was obtained from PhytoLab GmbH & Co. KG (Vestenbergsgreuth, Germany). The ultrapure water for the experiments was obtained from a Milli-Q water purification system Simplicity 185 by Millipore (Bedford, MA, USA).

### 3.3. Experimental Design, Process Range Conditions and Solvent Choice

As stated by Solarte et al. [36], the most relevant parameters affecting MAE are solvent/solid ratio, temperature, and treatment time. According to the preliminary single factor experiments (not reported) and literature data [16], solvent/solid ratio in the range from 10 to 30 mL/g, the temperature in the range from 80 to 120 °C and time in the range from 5 to 25 min were chosen for the establishing of Box–Behnken experimental design (BBD) (Table 6). Before carrying out the experimental design, the solvent choice was investigated. Sparr Eskilsson et al. [14] reported that water has a greater capacity to obstruct microwaves than organic solvents, which is based on the dielectric constant. Therefore, the 50% aqueous ethanol solution was selected as the extraction solvent in this study, considering its polar nature as well as the environmental acceptance.

The response surface methodology (RSM) was employed for the investigation of effects of MAE parameters and optimization of the conditions for various responses. BBD with three independent variables temperature (X_1_), time (X_2_) and solvent/solid ratio (X_3_) was used to determine the response pattern and to establish the models which will effectively describe the MAE process. The complete design consisted of 17 experiments for each type of waste and leaves as starting material with five replicates at the central point were included. 3D plots are generated using Design Expert^®^ commercial software (Ver. 9, Stat-Ease Inc., Minneapolis, MN, USA).

### 3.4. Procedure for Microwave-Assisted Extraction (MAE)

MAE of bioactive compounds was carried out following the experimental plan given in Table 6 with 50% aqueous ethanol solution as a solvent. Extraction was performed on a Milestone flexiWAVE Microwave apparatus using a closed-vessel system (Milestone Srl, Sorisole (BG), Italy) and setting the maximum microwave power at 700 W. In order to maintain the constant temperature, the power was not applied constantly during the experiment. Pre-heating time was set for 2 min and cooling time for 10 min. A light yellow to a dark brown solution was obtained after MAE. Then the vessels were cooled down to room temperature, evaporated on SpeedVac (SPD1030, Thermo Scientific, Waltham, MA, USA) and dry extracts were stored in the +4 °C until analyzed. The extraction yield was calculated using Equation (9) where W_1_ represents the mass of the obtained extract after solvent evaporation and W_2_ represent the mass of plant material (tobacco leaves and waste) used for the extraction process.

Y(%) = W_1_/W_2_ × 100 (9)

### 3.5. High Performance Liquid Chromatography (HPLC) Analysis

Identification and quantification of nicotine in tobacco leaves and waste extracts was done according to the isocratic method reported in study by Bansal et al. [37]) using Lab Poroshell 120 EC-C18 (4.6 × 150 mm, 4 µm) column and acetonitrile and carbonate buffer (pH 10, 0.03 M) as mobile phase.

Identification and quantification of phenolic compounds (CCA, NCA, CA, rutin and nicotiflorin) was performed using previously reported gradient method [38] with separation obtained on a: COSMOSIL 5C18-MS-11 (4.6 × 250 mm, 5 µm) chromatographic column (Agilent, Santa Clara, CA, USA) and using methanol and 1% acetic acid solution in Milli-Q water (conductivity ≤ 0.054 μS/cm) as a mobile phase.

Both HPLC analyses of phenolic compounds were performed on an Agilent 1260 Infinity II device (Agilent, Santa Clara, CA, USA), equipped with a quaternary pump a column chamber, a Variable Wavelength Detector (VWD) and an autosampler. Prior to analysis, extracts were diluted in the mobile phase and filtered through 0.2 µm polytetrafluoroethylene (PTFE) syringe filter. All measurements were performed in triplicate and results were expressed in weight percentage (% *w*/*w*). 

### 3.6. Spectrophotometric Assays

The antiradical activity expressed as percent of inhibition of DPPH radical (% DPPH) and total phenolic content (TPC) of MAE extracts were determined by spectrophotometric assays as explained in detail by Molnar et al. [39] and Jakobek et al. [40]. TPC results were expressed in weight percentage (% *w*/*w*) of gallic acid equivalents. All measurements were performed in triplicate.

### 3.7. Statistical Analysis

For statistical analysis software tools, Statistica 13.3 (TIBCO Software Inc., Palo Alto, CA, USA) was used at a significance level *p* = 0.05. Normality of data distribution was tested by the nonparametric Kolmogorov–Smirnov test for the comparison of medians and arithmetic mean. Parametric and nonparametric statistical tests were combined due to variable distribution of numerical variables. Numerical variables are shown as arithmetic means and standard deviation, along with minimum and maximum values or median and interquartile range. Besides descriptive statistical analysis and correlation calculations, a comparison of independent variables and the analysis of variance (ANOVA) test was used.

## 4. Conclusions

This work is a good example of valorization of three types of tobacco industrial waste using MAE with recovering of high added value compounds (nicotine and phenolic compounds). RSM was successfully applied for the description of variable behaviors, and optimization of the extraction process conditions. Obtained results for waste extracts, compared with leaves clarify the need for studies that provide detailed insight into the impact of process parameters on the composition of MAE extracts for every type of tobacco waste. In addition, based on experimental results can be concluded the MAE process allowed to obtain both, an extract rich in nicotine and an extract with lower amount of nicotine; thus, simplifying the waste management and increasing the efficiency of its potential use in other processes or products.

## Figures and Tables

**Table 1:** Chemical composition of crude tobacco leaves extracts obtained with MAE.

Run	Yield (%)	Nicotine (%)	DPPH (%)	TPC (%)	Individual Phenolic Compounds Concentration				
					CA (%)	Rutin (%)	NCA (%)	CCA (%)	Nicotiflorin (%)
1	57.700	2.442	72.611	4.144	0.966	0.327	0.409	0.432	0.067
2	52.320	4.781	75.168	3.933	0.939	0.256	0.279	0.168	0.063
3	44.080	5.095	69.128	3.733	1.423	0.161	0.291	0.399	nd
4	46.340	1.512	38.156	1.933	0.145	0.111	0.054	0.017	nd
5	60.780	3.565	76.350	4.000	1.143	0.399	0.344	0.401	0.072
6	49.860	4.799	80.761	4.078	1.501	0.492	0.323	0.413	0.072
7	48.120	5.106	75.328	3.800	0.979	0.250	0.244	0.229	0.044
8	46.001	5.480	76.926	4.000	0.864	0.371	0.226	0.161	nd
9	42.640	5.022	80.153	3.400	0.867	0.396	0.193	0.152	nd
10	55.680	3.066	53.404	3.000	0.321	0.314	0.105	0.095	nd
11	69.780	5.286	84.660	4.511	0.822	0.635	0.180	0.225	0.089
12	56.520	4.248	71.492	4.444	0.479	0.341	0.218	0.210	0.071
13	39.960	4.335	73.282	3.600	0.979	0.266	0.119	0.108	0.041
14	53.040	4.335	81.400	3.800	0.653	0.504	0.166	0.162	0.071
15	49.080	5.126	88.910	4.367	0.894	0.367	0.174	0.185	0.087
16	19.402	4.733	83.190	3.644	0.839	0.534	0.206	0.187	0.076
17	47.080	5.392	79.134	4.289	0.903	0.296	0.168	0.166	0.043

TPC—total phenol content, DPPH—antiradical activity, CA—chlorogenic acid, NCA—neochlorogenic acid, CCA—cryptochlorogenic acid, nd—not detected.

**Table 2:** Chemical composition of crude tobacco waste extracts (type: scrap) obtained with MAE.

Run	Yield (%)	Nicotine (%)	DPPH (%)	TPC (%)	Individual Phenolic Compounds Concentration				
					CA (%)	Rutin (%)	NCA (%)	CCA (%)	Nicotiflorin (%)
1	54.720	2.053	67.018	4.633	0.229	0.222	0.151	0.058	0.064
2	52.960	2.976	43.635	3.622	0.329	0.256	0.197	0.050	0.042
3	41.480	3.427	55.378	3.933	0.401	0.253	0.048	0.041	0.042
4	35.980	2.843	54.084	4.133	0.468	0.464	0.081	0.068	0.082
5	58.530	2.078	63.751	4.300	0.289	0.175	0.074	0.045	0.075
6	47.200	2.758	62.730	4.567	0.430	0.326	0.075	0.050	0.053
7	47.080	3.196	56.603	3.889	0.332	0.288	0.179	0.043	0.046
8	50.120	3.327	55.514	3.633	0.367	0.265	0.154	0.030	0.043
9	48.180	3.547	47.107	3.611	0.394	0.251	0.156	0.064	0.040
10	42.240	3.117	42.478	4.011	0.312	0.307	0.138	0.056	0.052
11	47.120	3.709	50.294	3.889	0.495	0.631	0.191	0.092	0.057
12	69.081	1.886	47.759	4.244	0.180	0.130	0.169	0.057	0.057
13	43.202	2.975	24.174	2.811	0.334	0.222	0.162	0.031	0.044
14	43.960	3.115	66.351	4.711	0.525	0.469	0.256	0.136	0.083
15	42.740	3.450	57.849	4.578	0.257	0.353	0.183	0.039	0.056
16	46.380	3.330	50.038	3.611	0.449	0.312	0.188	0.082	0.050
17	45.000	2.870	43.380	2.756	0.417	0.256	0.188	0.041	0.043

TPC—total phenol content, DPPH—antiradical activity, CA—chlorogenic acid, NCA—neochlorogenic acid, CCA—cryptochlorogenic acid.

**Table 3:** Chemical composition of crude tobacco waste extracts (type: dust) obtained with MAE.

Run	Yield (%)	Nicotine (%)	DPPH (%)	TPC (%)	Individual Phenolic Compounds Concentration				
					CA (%)	Rutin (%)	NCA (%)	CCA (%)	Nicotiflorin (%)
1	49.640	2.720	14.571	1.397	0.273	0.126	0.181	0.028	0.040
2	38.560	3.270	21.793	1.840	0.880	0.514	0.226	0.066	0.090
3	35.640	3.717	23.867	1.963	0.584	0.568	0.228	0.074	0.099
4	42.920	3.505	18.335	1.480	0.425	0.313	0.177	0.038	0.050
5	58.230	2.628	12.266	1.448	1.112	0.284	0.188	0.030	0.050
6	37.820	3.930	26.658	2.027	0.454	0.501	0.235	0.059	0.088
7	37.800	4.064	30.166	1.928	0.952	0.496	0.232	0.062	0.086
8	47.960	3.238	36.389	2.257	1.019	0.505	0.245	0.116	0.070
9	48.075	3.467	9.040	2.196	0.932	0.534	0.344	0.117	0.074
10	47.520	4.840	12.343	2.763	0.945	0.218	0.119	0.012	0.039
11	39.080	3.349	44.123	2.182	0.237	0.422	0.219	0.066	0.079
12	52.880	2.654	19.565	1.922	0.484	0.180	0.274	0.219	0.041
13	38.320	3.405	31.396	2.336	0.737	0.602	0.245	0.068	0.095
14	46.440	3.293	26.709	2.277	0.546	0.333	0.203	0.036	0.051
15	29.420	3.408	27.478	2.231	1.135	0.418	0.236	0.249	0.073
16	40.680	3.196	32.241	2.657	0.473	0.534	0.245	0.243	0.092
17	38.960	3.169	31.396	2.697	0.931	0.485	0.252	0.073	0.085

TPC—total phenol content, DPPH—antiradical activity, CA—chlorogenic acid, NCA—neochlorogenic acid, CCA–cryptochlorogenic acid.

**Table 4:** Chemical composition of crude tobacco waste extracts (type: midrib) obtained with MAE.

Run	Yield (%)	Nicotine (%)	DPPH (%)	TPC (%)	Individual Phenolic Compounds Concentration				
					CA (%)	Rutin (%)	NCA (%)	CCA (%)	Nicotiflorin (%)
1	49.000	1.503	30.960	1.552	0.101	0.103	0.077	0.001	0.037
2	52.760	1.310	29.642	1.054	0.192	0.100	0.069	nd	nd
3	49.280	1.656	23.352	1.280	0.153	0.104	0.056	nd	nd
4	42.520	1.619	59.896	2.806	0.014	0.166	0.323	0.444	0.075
5	57.960	1.325	32.553	1.288	0.185	0.099	0.081	0.001	nd
6.	37.840	1.379	40.716	1.484	0.250	0.140	0.090	0.009	nd
7	47.600	1.543	38.173	1.328	0.193	0.099	0.076	0.242	nd
8	51.440	1.322	45.969	2.637	0.193	0.106	0.064	nd	0.073
9	53.100	1.323	44.296	1.266	0.208	0.119	0.070	nd	nd
10	47.160	0.867	33.322	1.302	0.212	0.113	0.056	nd	nd
11	43.600	1.628	48.652	1.393	0.190	0.094	0.055	nd	nd
12	52.400	1.247	31.014	1.713	0.113	0.096	0.090	nd	0.074
13	49.600	1.403	45.902	2.294	0.188	0.095	0.068	nd	nd
14	49.160	1.272	42.623	2.261	0.202	0.120	0.088	nd	nd
15	41.900	1.783	36.132	1.479	0.266	0.141	0.086	nd	nd
16	51.720	1.442	42.489	1.274	0.228	0.098	0.076	nd	nd
17	50.400	1.431	43.961	1.238	0.211	0.115	0.074	nd	nd

TPC—total phenol content, DPPH—antiradical activity, CA—chlorogenic acid, NCA—neochlorogenic acid, CCA—cryptochlorogenic acid, nd—not detected.

**Table 5:** Optimal MAE conditions for tobacco leaves and tobacco waste obtained by response surface methodology.

	Leaves	Scrap	Dust	Midrib
Criteria: Maximum extraction yield with maximum nicotine content				
Temperature (°C)	80.0	80.0	80.31	80.0
Time (min)	5.0	5.0	5.0	25.0
Solvent/solid ratio (mL/g)	26.1	29.8	30.01	30.00
Predicted yield value (%)	68.624	49.868	48.888	53.455
Predicted nicotine value (%)	5.638	3.268	4.058	1.603
Criteria: Maximum extraction yield with minimum nicotine content				
Temperature (°C)	120.0	120.0	119.3	120.0
Time (min)	25.0	25.0	19.4	11.2
Solvent/solid ratio (mL/g)	10.0	19.1	30.0	30.0
Predicted yield value (%)	66.123	68.315	58.233	55.961
Predicted nicotine value (%)	2.311	1.870	2.628	1.045

**Table 6:** BBD experimental design for MAE of bioactive compounds from tobacco leaves and waste.

Run	Temperature (°C)	Time (min)	Solvent/Solid Ratio (mL/g)
1	120	15	10
2	100	15	20
3	80	25	20
4	80	15	10
5	120	15	30
6	100	25	10
7	100	15	20
8	100	15	20
9	80	15	30
10	100	5	30
11	80	5	20
12	120	25	20
13	100	15	20
14	120	5	20
15	100	5	10
16	100	25	30
17	100	15	20

## Data Availability

The data presented in this study are available on request from the corresponding author.

## Supplementary Materials

The following are available online, Figure S1: Comparison of applied microwave power dependent on MAE parameters on two temperatures: (a) 80 °C and (b) 120 °C. Table S1: Spearman’s Rank Order Correlations between yield per every sample and particular components in tobacco leaves and waste extracts. Table S2: Comparison in yield, particular content of compounds, antiradical activity (DPPH), and total phenolic content (TPC) in tobacco leaves and waste extracts.

## Sample Availability

Samples of the compounds are available on request from the corresponding authors.

## References

[1] Banožić M., Babić J., Jokić S. (2020). Recent advances in extraction of bioactive compounds from tobacco industrial waste-a review. Ind. Crop. Prod..

[2] Wang J., Lu D., Zhao H., Jiang B., Wang J., Ling X., Chai H., Ouyang P. (2010). Discrimination and classification of tobacco wastes by identification and quantification of polyphenols with LC–MS/MS. J. Serb. Chem. Soc..

[3] Rincón J., De Lucas A., García M.A. (1998). Preliminary study on the supercritical carbon dioxide extraction of nicotine from tobacco wastes. Sep. Sci. Technol..

[4] Hu R.S., Wang J., Li H., Ni H., Chen Y.-F., Zhang Y.W., Xiang S.-P., Li H.H. (2015). Simultaneous extraction of nicotine and solanesol from waste tobacco materials by the column chromatographic extraction method and their separation and purification. Sep. Purif. Technol..

[5] Wang W., Wang Y., Yang L., Liu B., Lan M., Sun W. (2005). Studies on thermal behavior of reconstituted tobacco sheet. Thermochim. Acta.

[6] Wang Y., Gu W. (2018). Study on supercritical fluid extraction of solanesol from industrial tobacco waste. J. Supercrit. Fluid..

[7] Jokić S., Gagić T., Knez Ž., Banožić M., Škerget M. (2019). Separation of active compounds from tobacco waste using subcritical water extraction. J. Supercrit. Fluid..

[8] Banožić M., Jozinović A., Grgić J., Miličević B., Jokić S. (2021). High voltage electric discharge for recovery of chlorogenic acid from tobacco waste. Sustainability.

[9] Civilini M., Domenis C., Sebastianutto N., Bertoldi M. (1997). Nicotine decontamination of tobacco agro-industrial waste and its degradation by microorganisms. Waste Manag. Res..

[10] Briški F., Horgas N., Vuković M., Gomzi Z. (2003). Aerobic composting of tobacco industry solid waste—simulation of the process. Clean Technol. Environ. Policy.

[11] Cvjetko Bubalo M., Vidović S., Radojčić Redovniković I., Jokić S. (2015). Green solvents for green technologies. J. Chem. Technol. Biotechnol..

[12] González A.M., Barnes R.M. (2002). Comparison of microwave-assisted extraction and waste extraction test (WET) preparation for inductively coupled plasma spectroscopic analyses of waste samples. Anal. Bioanal. Chem..

[13] Belwal T., Chemat F., Venskutonis P.R., Cravotto G., Jaiswal D.K., Bhatt I.D., Devkota H.P., Luo Z. (2020). Recent advances in scaling-up of non-conventional extraction techniques: Learning from successes and failures. TrAC.

[14] Eskilsson C.S., Björklund E. (2000). Analytical-scale microwave-assisted extraction. J. Chromatogr. A.

[15] Jassie L., Revesz R., Kierstead T., Hasty E., Metz S., Kingston H.M., Haswell S.J. (1997). Microwave-assisted extraction of polysaccharides. Microwave-Enhanced Chemistry.

[16] Chaturvedi A.K. (2018). Extraction of neutraceuticals from plants by microwave assisted extraction. Sys. Rev. Pharm..

[17] Garcia-Vaquero M., Ummat V., Tiwari B., Rajauria G. (2020). Exploring ultrasound, microwave and ultrasound–microwave assisted extraction technologies to increase the extraction of bioactive compounds and antioxidants from brown macroalgae. Mar. Drugs.

[18] Bagade S.B., Patil M. (2019). Recent advances in microwave assisted extraction of bioactive compounds from complex herbal samples: A review. Crit. Rev. Anal. Chem..

[19] Zhu X., Su Q., Cai J., Yang J. (2006). Optimization of microwave-assisted solvent extraction for volatile organic acids in tobacco and its comparison with conventional extraction methods. Anal. Chim. Acta.

[20] Joners N.M., Bernardo-Gil M.G., Lourenço M.G. (2001). Comparison of methods for extraction of tobacco alkaloids. J. AOAC Int..

[21] Nie J., Yu G., Song Z., Wang X., Li Z., She Y., Lee M. (2017). Microwave-assisted deep eutectic solvent extraction coupled with headspace solid-phase microextraction followed by GC-MS for the analysis of volatile compounds from tobacco. Analytical Methods.

[22] Zhou H.Y., Liu C.Z. (2006). Microwave-assisted extraction of solanesol from tobacco leaves. J. Chromatogr. A.

[23] Machado P.A., Fu H., Kratochvil R.J., Yuan Y., Hahm T.S., Sabliov C.M., Wei C., Lo Y.M. (2010). Recovery of solanesol from tobacco as a value-added byproduct for alternative applications. Bioresour. Technol..

[24] Li Z., Huang D., Tang Z., Deng C., Zhang X. (2010). Fast determination of chlorogenic acid in tobacco residues using microwave-assisted extraction and capillary zone electrophoresis technique. Talanta.

[25] Banožić M., Gagić T., Čolnik M., Knez Ž., Škerget M., Jerković I., Jokić S. (2021). Sequence of supercritical CO_2_ extraction and subcritical H_2_O extraction for the separation of tobacco waste into lipophilic and hydrophilic fractions. Chem. Eng. Res. Des..

[26] Banožić M., Banjari I., Jakovljević M., Šubarić D., Tomas S., Babić J., Jokić S. (2019). Optimization of ultrasound-assisted extraction of some bioactive compounds from tobacco waste. Molecules.

[27] Docheva M.H., Kochev Y.G., Kirkova D.M., Stoilova A.B. (2020). Antioxidant activity and chemical composition of crude extracts from different tobaccos and tobacco blends. Bulg. Chem. Commun..

[28] Tayoub G., Sulaiman H., Alorfi M. (2015). Determination of nicotine levels in the leaves of some *Nicotiana tabacum* varieties cultivated in Syria. Herba Pol..

[29] Popova V., Ivanova T., Stoyanova A., Georgiev V., Hristeva T., Nikolova V., Docheva M., Nikolov N., Damianova S. (2018). Phytochemicals in leaves and extracts of the variety “Plovdiv 7” of Bulgarian oriental tobacco (*Nicotiana tabacum* L.). Trends Phytochem. Res..

[30] Popova V.T., Ivanova T.A., Stoyanova A.S., Nikolova V.V., Docheva M.H., Hristeva T.H., Nikolov N.P. (2020). Chemical constituents in leaves and aroma products of *Nicotiana rustica* L. tobacco. Int. J. Food Stud..

[31] Chen Y., Jimmy Y.Q., Li X., Luo Y., Liu H. (2007). Extraction and HPLC characterization of chlorogenic acid from tobacco residuals. Sep. Sci. Technol..

[32] Kamaruddin M.J., Hamid S.R.A., Othman S.I.A., Alam M.N.H.Z., Zaini M.A.A., Zakaria Z.Y. (2018). The effects of conventional and microwave heating techniques on extraction yield of *Orthosiphon stamineus* leaves. Chem. Eng. Trans..

[33] Hu F., Deng C., Liu Y., Zhang X. (2009). Quantitative determination of chlorogenic acid in Honeysuckle using microwave-assisted extraction followed by nano-LC-ESI mass spectrometry. Talanta.

[34] Alara O.R., Abdurahman N.H., Olalere O.A. (2018). Optimization of microwave-assisted extraction of flavonoids and antioxidants from *Vernonia amygdalina* leaf using response surface methodology. Food Bioprod. Process..

[35] Butorac J. (2009). Duhan.

[36] Solarte D.A., Ruiz-Matute A.I., Chito-Trujillo D.M., Rada-Mendoza M., Sanz M.L. (2021). Microwave assisted extraction of bioactive carbohydrates from different morphological parts of alfalfa (*Medicago sativa* L.). Foods.

[37] Bansal M., Sharma M., Bullen C., Svirskis D. (2018). A stability indicating HPLC method to determine actual content and stability of nicotine within electronic cigarette liquids. Int. J. Environ. Res. Public Health.

[38] Šafranko S., Ćorković I., Jerković I., Jakovljević M., Aladić K., Šubarić D., Jokić S. (2021). Green extraction techniques for obtaining bioactive compounds from mandarin peel (*Citrus unshiu* var. *Kuno*): Phytochemical analysis and process optimization. Foods.

[39] Molnar M., Jerković I., Suknović D., Bilić Rajs B., Aladić K., Šubarić D., Jokić S. (2017). screening of six medicinal plant extracts obtained by two conventional methods and supercritical CO_2_ extraction targeted on coumarin content, 2,2-Diphenyl-1-picrylhydrazyl radical scavenging capacity and total phenols content. Molecules.

[40] Jakobek L., Šeruga M., Novak I., Medvidović-Kosanović M. (2007). Flavonols, phenolic acids and antioxidant activity of some red fruits. Dtsch. Lebens. Rundsch..

