# Supplementary material for: Optimization of MAE for the Separation of Nicotine and Phenolics from Tobacco Waste by Using the Response Surface Methodology Approach
Source: Molecules. 2021 Jul 19;26(14):4363. doi: 10.3390/molecules26144363 (PMC8303117; doi:10.3390/molecules26144363)
Supplement: Supplementary file 1 [file molecules-26-04363-s001.zip › molecules-1306148-supplementary.pdf]

molecules

MDPI

Supplementary Materials

Optimization of MAE for the Separation of Nicotine and Phenolics from Tobacco Waste by Using the Response Surface Methodology Approach

Marija Banožić [1], Ines Banjari [1], Ivana Flanjak [1], Mate Paštar [2], Jelena Vladić [3,*] and Stela Jokić [1,*]

[1] Faculty of Food Technology Osijek, Josip Juraj Strossmayer University of Osijek, Franje Kuhača 18, 31000 Osijek, Croatia; marija.banozic@ptfos.hr (M.B.); ines.banjari@ptfos.hr (I.B.); ivana.flanjak@ptfos.hr (I.F.)
[2] Public Institution RERA S.D. for Coordination and Development of Split-Dalmatia County, Domovinskog rata 2, 21000 Split, Croatia; mate.pastar@rera.hr
[3] Faculty of Technology, University of Novi Sad, Bulevar cara Lazara 1, 21 000 Novi Sad, Serbia
* Correspondence: vladicj@uns.ac.rs (J.V.); stela.jokic@ptfos.hr (S.J.);
Tel.: + 381-21-485-3728 (J.V.); +385-31-224-333 (S.J.)

Table S1. Spearman's Rank order correlations between yield per every sample and particular components in tobacco leaves and waste extracts

Yield	Nicotine	CA	NCA	CCA	Nicotiflorin	Rutin	DPPH	TPC
Leaves	-0.275	-0.100	0.120	0.419	0.520*	0.265	-0.032	0.568*
Scrap	-0.358	-0.434	0.064	0.070	0.028	-0.530*	0.147	0.067
Dust	-0.566*	-0.066	-0.096	-0.265	-0.718*	-0.539*	-0.471	-0.272
Midrib	-0.574*	-0.370	0.164	-0.180	-0.524*	-0.157	0.199	0.152
CA- chlorogenic acid, NCA-neochlorogenic acid, CCA- cryptochlorogenic acid, TPC- total phenol content, DPPH- antiradical activity								

*statistically significant at $p<0.05$

Table S2. Comparison in yield, particular content of compounds, antiradical activity (DPPH) and total phenolic content (TPC) in tobacco leaves and waste extracts

	Leaves	Dust	*p*	Scrap	*p*	Midrib	*p*
Yield	49.3 ± 10.7	40.68 (38.32 - 47.96)	0.017*	47.08 (43.20 - 50.12)	0.344	40.68 (38.32 - 47.96)	0.017*
Nicotine	4.78 (4.25 – 5.11)	3.349 (3.196 - 3.505)	0.003*	2.980 ± 0.533	<0.001*	1.415 ± 0.208	<0.001*
CA	0.894 (0.822–0.979)	0.737 (0.473 - 0.945)	0.286	0.365 ± 0.095	<0.001*	0.193 (0.185 - 0.211)	<0.001*
NCA	0.229 ± 0.087	0.232 (0.203 - 0.245)	0.836	0.162 (0.138 - 0.188)	0.003*	0.077 (0.070 - 0.090)	0.005*
CCA	0.185 (0.161 – 0.229)	0.066 (0.038 - 0.116)	0.002*	0.050 (0.041 - 0.064)	<0.001*	102.000 (0.444 - 102.000)	0.002*
Nicotiflorin	0.003 (0.002 – 0.006)	0.074 (0.050 - 0.088)	<0.001*	0.052 (0.043 - 0.057)	<0.001*	102.000 (102.000 - 102.000)	<0.001*
Rutin	0.354 ± 0.134	0.485 (0.313 - 0.514)	0.168	0.265 (0.251 - 0.326)	0.129	0.104 (0.099 - 0.119)	<0.001*
DPPH	76.414 (72.675 – 81.304)	23.578 ± 8.058	<0.001*	52.415 ± 10.705	<0.001*	40.938 ± 10.527	<0.001*
TPC	3.933 (3.644 – 4.144)	2.071± 0.503	<0.001*	3.937 ± 0.572	0.605	1,393 (1.280 – 1.713)	<0.001*
CA- chlorogenic acid, NCA-neochlorogenic acid, CCA- cryptochlorogenic acid, TPC- total phenol content, DPPH- antiradical activity							

Mann-Whitney U test; *significant at $p<0.05$

a)

b)

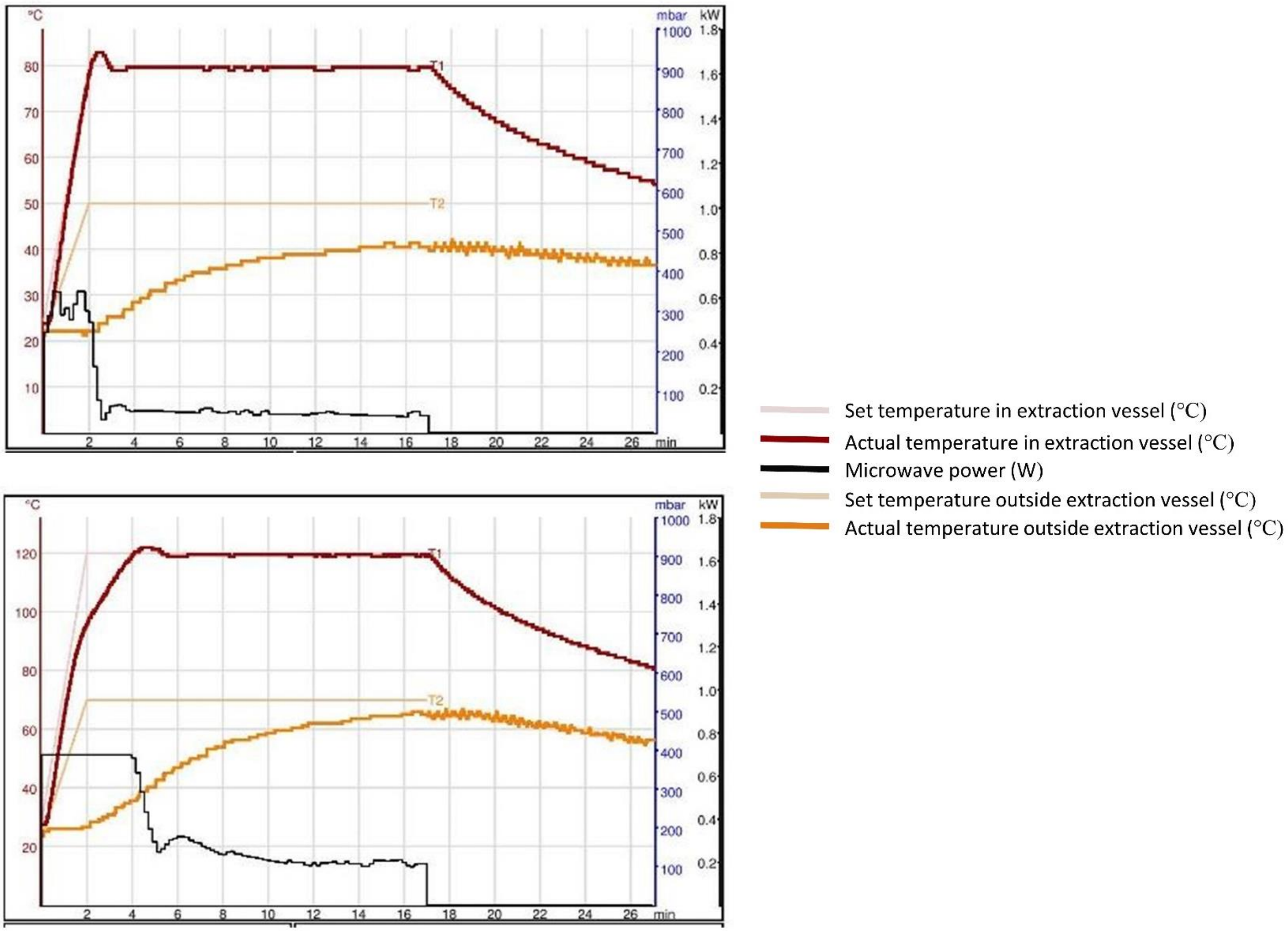
°C
mbar
kW
T1
T2
min
Set temperature in extraction vessel (°C)
Actual temperature in extraction vessel (°C)
Microwave power (W)
Set temperature outside extraction vessel (°C)
Actual temperature outside extraction vessel (°C)
